# Exploring the Views of Young People, Including Those With a History of Self-Harm, on the Use of Their Routinely Generated Data for Mental Health Research: Web-Based Cross-Sectional Survey Study

**DOI:** 10.2196/60649

**Published:** 2025-03-12

**Authors:** Dana Dekel, Amanda Marchant, Marcos Del Pozo Banos, Mohamed Mhereeg, Sze Chim Lee, Ann John

**Affiliations:** 1 Swansea University Medical School Swansea University Swansea United Kingdom

**Keywords:** self-harm, mental health, big data, survey, youth

## Abstract

**Background:**

Secondary use of routinely collected health care data has great potential benefits in epidemiological studies primarily due to the large scale of preexisting data.

**Objective:**

This study aimed to engage respondents with and without a history of self-harm, gain insight into their views on the use of their data for research, and determine whether there were any differences in opinions between the 2 groups.

**Methods:**

We examined young people’s views on the use of their routinely collected data for mental health research through a web-based survey, evaluating any differences between those with and without a history of self-harm.

**Results:**

A total of 1765 respondents aged 16 to 24 years were included. Respondents’ views were mostly positive toward the use and linkage of their data for research purposes for public benefit, particularly with regard to the use of health care data (mental health or otherwise), and generally echoed existing evidence on the opinions of older age groups. Individuals who reported a history of self-harm and subsequently contacted health services more often reported being “extremely likely” or “likely” to share mental health data (contacted: 209/609, 34.3%; 95% CI 28.0-41.2; not contacted: 169/782, 21.6%; 95% CI 15.8-28.7) and physical health data (contacted: 117/609, 19.2%; 95% CI 12.7-27.8; not contacted: 96/782, 12.3%; 95% CI 6.7-20.9) compared with those who had not contacted services. Respondents were overall less likely to want to share their social media data, which they considered to be more personal compared to their health care data. Respondents stressed the importance of anonymity and the need for an appropriate ethical framework.

**Conclusions:**

Young people are aware, and they care about how their data are being used and for what purposes, irrespective of having a history of self-harm. They are largely positive about the use of health care data (mental or physical) for research and generally echo the opinions of older age groups raising issues around data security and the use of data for the public interest.

## Introduction

### Background

The era of “big data” has offered our current digital economy different perspectives across a variety of areas, bringing together epidemiologists, computer scientists, economists, sociologists, bioinformaticians, and other scholars with large volumes of data and information, produced by and about people. Researchers are accessing routinely collected data and then aggregating and linking those data across sectors (eg, business, science, justice, health, and social care), each with its own advantages, limitations, and ethical issues [1]. Secondary use of routinely collected health care data holds great potential benefits in epidemiological studies primarily due to the large scale of preexisting data [2]. This use of routinely collected health care data to inform policy allows evidence to be collected without placing a burden on individuals. This is of particular importance when looking to tailor support for individuals who may face stigma or other barriers to taking part in research, such as individuals with a history of self-harm (SH). The COVID-19 pandemic exemplified the importance of using health care data to inform policy and practice.

Big data lies at the heart of efforts to aggregate information that will assist in identifying disparate needs across geographic regions, groups, as well as epidemiological trends [3]. The advantages of routinely collected data need to be balanced against ethical and legal challenges, such as patient anonymity, data security, fairness, and transparency [4]. Health care researchers identified the need to gain a better understanding of the public’s attitudes toward the use of their data by enhancing their participation in the research process and ensuring use of their data aligns with public interests [5]. While the public is generally supportive of the use of their health care data [4,6-8], certain fields, such as mental health data, arguably remain differentiated due to their perceived sensitive nature [9] and have unique ethical considerations [10]. Studies exploring public views on sharing mental health data found community attitudes to be largely positive [9,11-13]. The need to question the long-term efficacy of measures used, the validity of the types of data resources, and the risks of bias and privacy were highlighted [10,13-17].

In contrast to health data, social media and other internet-based platforms often lack clear and consistent procedures for the use of data generated online [18]. This rapidly growing technological landscape is changing our definition of knowledge and raising significant ethical questions, namely “Can data be used simply because it is openly accessible?” as well as critically evaluating the methods of collection and biases associated with it [19]. Guidelines have been developed outlining ethical factors, such as confidentiality, security, consent, potential harm, and levels of researcher control, when engaging with internet-mediated research [20]. While there is much debate surrounding the ethical principles that need to be applied in social media or internet-based research [18,21-23], these often do not involve the views of online users or make efforts to improve their level of awareness as to what is or can be done with their data [24].

Young people are particularly susceptible to both generating and sharing vast amounts of personal data, to the point of being characterized as a valuable currency in the digital economy [25]. Online presence within this age group as well as its forms changed over time from being more text based to increasingly changing into communication through imagery and videos [25], including sharing images of SH [26,27]. Contingent on appropriately securing and ethically handling these types of data footprints, they can arguably be used to identify patterns and risk indicators of those who struggle with mental health and would potentially benefit from early interventions that may prevent SH and suicide [28,29].

There is a wide misplaced acceptance that young people’s willingness to heavily engage with online platforms suggests that they do not care about their privacy [30]. However, some authors advocate little variance between adults and young people when it comes to their views on privacy in data sharing [31-33]. Researchers have used online platforms to examine the mental health and well-being of young people across various topics [34,35]; however, young people’s views on the use of their data for mental health research purposes, though somewhat explored [36], remain underreported. Moreover, the views of young people who have experienced SH have not been examined previously in relation to the use of their data for research. The often hidden and stigmatized nature of SH may give rise to specific concerns from young people that have implications for data management and ethical use.

### This Study

Engagement with young people is essential to develop a better understanding of these issues and their views and was a key focus of this study. This survey set out to engage respondents with and without a history of SH in order to gain insights into their views and knowledge of how different kinds of data are anonymized and used for research and to determine whether there are any differences of opinions between those 2 groups. We also aimed to explore differences between those who had contacted health services for SH and those who had not.

## Methods

### Study Design

This study was a web-based cross-sectional survey. The Checklist for Reporting Results of Internet E-Surveys was followed [37].

### Ethical Considerations

Ethics approval was granted by the Swansea University Medical School Ethics Committee (2018-0062). Respondents gave informed consent, were made aware that participation in the survey was entirely voluntary and no incentives were offered. Data were given anonymously with no personally identifiable information collected. Any potentially identifiable information given in free-text responses was removed before analysis (see details in Data Analysis section).

### Description of Respondents

Respondents aged 16 to 24 years and residing in the United Kingdom were eligible. The survey was open to individuals both with and without a history of SH. SH is defined as intentional self-poisoning or self-injury, irrespective of the nature of motivation or degree of suicidal intent. This definition is necessarily very general because the nature of motivation or degree of suicidal intent is complex and may vary over time or between individuals [38]. A history of SH was based on self-report from respondents at the onset of the survey.

Recruitment occurred through a social media campaign conducted on Facebook (Meta platforms, Inc) and Instagram (Meta platforms, Inc) using visual material (eg, video clips and posters). Respondents were targeted based on age and location. Participants were also recruited via the Self-Harm Research UK research register. This is a register of individuals who have consented to be contacted for SH research [39].

### Data Collection

Responses were collected between July 18, 2019, and January 31, 2020. The survey (including respondent information, consent, and debrief forms) was hosted on Survey Monkey (SurveyMonkey Inc) [40] due to its user-friendly interface and its capability to record anonymous responses. SurveyMonkey uses a unique user identifier based on cookies to ensure each respondent takes the survey only once.

### Measures

#### Overview

The big data and mental health research survey consisted of 4 topic areas: demographic information, preferences and experiences of mental health and SH-based research, use of artificial intelligence, and big data for mental health research (a copy of the questionnaire can be found in Multimedia Appendix 1). There were 29 questions developed with student interns and piloted with a small group of participants from the Self-Harm Research UK research register. A subset of items was duplicated in both our survey and another survey exploring views on the use of data in a sample of National Health Service (NHS) patients [12]. This was done to allow meaningful comparison between the 2 populations. All items were optional except those collecting demographic information. For the purpose of this study, the items mentioned in the subsequent subheadings were used.

#### Demographic Information

In total, 4 mandatory questions related to age, gender, location, and history of SH were included. There were 3 additional optional questions related to SH including frequency.

#### Big Data for Mental Health Research

This section started with a brief description of what was meant by big data and how routinely collected data are collected, anonymized, and used for research, followed by 12 questions (5 closed-ended questions and 7 questions with free text responses questions; refer to survey questionnaire in Multimedia Appendix 1).

### Data Analysis

#### Overview

SurveyMonkey collects data from all respondents independent of the number of items completed. Eligibility for inclusion in data extraction based on item completion was determined retrospectively by the research team. It was decided a priori that respondents who answered all 4 demographic questions and a minimum of 2 other survey questions would be included. Respondents who chose “prefer not to answer” when asked about SH were excluded.

Respondents were stratified into 2 groups as follows: those reporting a history of SH and those reporting no history of SH (NoSH). Within the SH group individuals were further stratified into those who have made contact with health services following SH and those who have not. Health services were defined as general practitioners (GPs), any hospital treatment (eg, accident and emergency), and psychiatric or mental health services, including liaison services. Due to small numbers, it was not possible to break down service contacts by type (eg, GP).

#### Analysis of Quantitative Data

Descriptive statistics were used to summarize group characteristics and proportions of respondents across closed questions (counts, percentages, and 95% CIs). Wilson score with continuity correction was used to calculate CIs [41]. Cross-tabulation, together with chi-square test of association, was used to assess the significance of the difference in distributions between groups. We adopted available case analysis and performed pairwise deletion of nonresponses for each cross-tabulation. Chi-square tests were conducted to investigate the number of nonresponses for each item by age, gender, SH (NoSH vs SH group), contact with health services following SH. We did not correct for running multiple comparisons (eg, Bonferroni) when analyzing the quantitative data. This decision was taken due to the exploratory nature of the work to allow the exploration of more subtle effects in the data. The level of statistical significance was set at *P*=.05, and all statistical analyses were performed with SPSS (version 26, IBM Corp).

#### Extraction of Qualitative Data and Free Text Responses

To analyze responses to open-ended questions, identifiable information (eg, names and locations) were removed from any quotations and replaced with a random initial. A dualistic thematic analysis technique was used, using a deductive and inductive approach informed by the work of Fereday and Muir-Cochrane [42]. The deductive component involved creating a preliminary codebook to help guide the analysis, which was based on the open-ended questions asked in the survey as well as an initial scan of the quantitative responses. This was followed by facilitating an inductive generation of codes that allowed for unexpected themes to develop during the coding process [43]. For the latter, 2 members of the study team (DD and AM) independently identified recurrent and prominent themes in an initial sample of responses (1071/10,710, 10% of each item) for discussion. Codes were further reviewed and discussed with a third expert researcher (AJ). New codes were added, and themes were consolidated. The coding frame was piloted on 20% (2142/10,710) of the responses by 2 independent coders. Interrater agreement was assessed by computing Cohen κ coefficient. Codes were further discussed and refined, and sources of disagreement were resolved. Following this, items were recoded and interrater agreement was recalculated. All items included in the final coding frame had a Cohen κ score ≥0.7 [44].

Respondent responses for each free text question were broadly divided into “positive or yes”; “negative or no”; “neutral, unsure, or unclear” (eg, “don’t know” and “bleh”); and “conditional” indicating that the individuals’ response would be conditional on other factors.

Free text responses were organized and analyzed using NVivo (version 22, Lumivero). Cohen κ coefficients were calculated using Microsoft Excel.

## Results

### Respondents

A total of 3700 individuals took part in the survey. On the basis of the inclusion criteria, 1765 (47.7%) respondents were included in the analysis. Respondents gave 10,710 responses to 7 open-ended questions. Supplementary analysis was conducted to examine differences in item completion rates across questions and demographics (Multimedia Appendix 2). Nonresponses made up less ≤6% for each closed-ended question. Participants with a gender other than man/boy, woman/girl, older age, a history of SH, and those having contact with services were less likely to skip individual items.

We stratified participants into 2 groups based on the presence (SH) or absence (NoSH) of a history of SH. Of the 1765 included in the analysis 1448 (82.04%; 95% CI 79.9-83.9) reported previous SH. The demographics of respondents are shown in Table 1. Individuals aged 20 to 24 years were grouped together due to a small sample size.

**Table 1:** Demographics of respondents included in data extraction (N=1765).

Demographic characteristics			Self-harm (n=1448), n (%; 95% CI)		No self-harm (n=317), n (%; 95% CI)		Total (N=1765), n (%; 95% CI)
**Gender**							
	Female	1271 (87.8; 85.8-89.5)		281 (88.6; 84.2-90.3)		1552 (87.9; 86.2-89.5)	
	Male	87 (6; 2.3-13.8)		33 (10.4; 3-19.3)		120 (6.8; 3.2-13.3)	
	Other or unknown	90 (6.2; 2.5-13.9)		3 (0.9; 2.7-6.6)		93 (5.3; 1.9-12.5)	
**Age (y)**							
	16	699 (48.3; 44.5-52)		175 (55.2; 47.5-58.9)		874 (49.5; 46.2-52.9)	
	17	469 (32.4; 28.2-36.9)		81 (25.6; 16.8-29.8)		550 (31.2; 27.3-35.2)	
	18	86 (5.9; 2.2-13.8)		9 (2.8; 0.2-9.7)		95 (5.4; 2-12.6)	
	19	58 (4; 0.8-13.7)		9 (2.8; 0.2-12.1)		67 (3.8; 0.9-12.4)	
	20-24	136 (9.4; 5.3-15.9)		43 (13.6; 5.6-20.7)		179 (10.1; 6.3-15.8)	

A total of 41.02% (594/1448; 95% CI 37.0-45.1) of those who reported SH did so with suicidal intent. Self-injury (1370/1448, 94.61%; 95% CI 93.2-95.7) was the most frequently used method.

This was followed by ingesting medication in excess of the prescribed dose (570/1448, 39.36%; 95% CI 35.4-43.6).

When asked whether help was sought following incidents of SH, 39.71% (575/1448; 95% CI 35.7-43.8) of the respondents reported not seeking help from any of the sources listed. Friends, family, and neighbors were the most common sources of help (555/1448, 38.33%; 95% CI 34.3-42.5) followed by psychiatric or mental health services.

Among the 1448 respondents in the SH group, 42.06% (609/1448; 95% CI 38.2-46.1) reported contacting health services following an incident of SH. Of the 609 respondents who contacted health services, psychiatric or mental health services (n=518, 85.1%; 95% CI 82.0-87.8) were the most frequently reported followed by GPs (n=346, 56.8%; 95% CI 52.8-60.8) and hospital treatment (n=240, 39.4%; 95% CI 35.5-43.4).

### Topic Areas

#### Overview

Results of both closed- and open-ended questions are presented subsequently and organized by topic area (sharing types of data research, use of mental health data, factors influencing feelings toward data use, trustworthiness of organizations, and the use of anonymized health care data). Responses from the SH and NoSH group were broadly similar. Consequently, we only highlighted group differences wherever necessary.

#### Topic 1: Sharing of Types of Data for Research

Respondents were asked the question, “Thinking about data more generally (not only health data), how likely would you be to share the following types of data for research (ethnicity, marital status, social media, physical health, mental health, etc)?”

**Figure 1:**
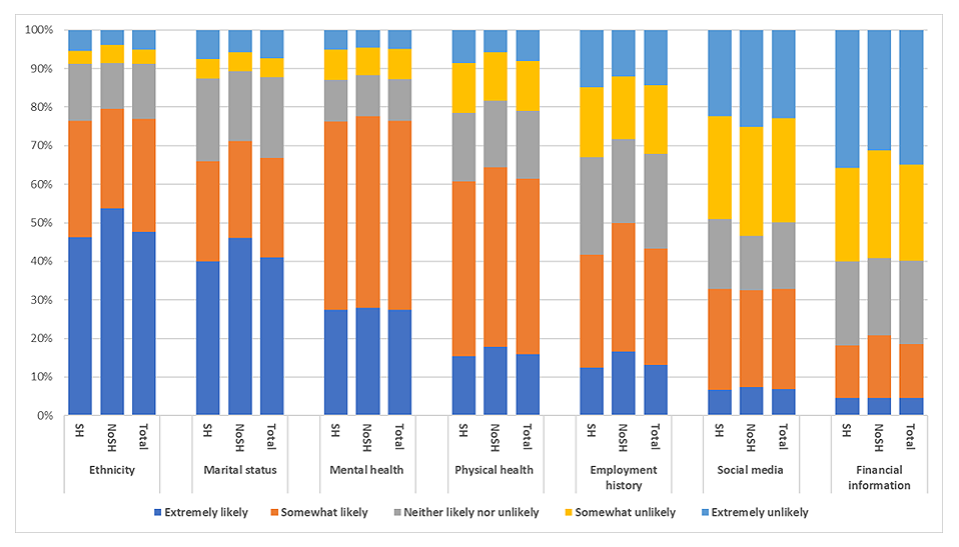
Responses to the question “Thinking about data more generally (not only health data), how likely would be to share various types of data for research purposes?” NoSH: no history of self-harm; SH: history of self-harm.

Respondents were more likely to share their ethnicity, marital status, and mental and physical health data and less likely to share their social media posts and financial information. There were no substantial differences between the NoSH and the SH groups in the distributions of responses across all types of data (Figure 1, Multimedia Appendix 3). The NoSH group showed a slightly higher likelihood of sharing their data through most categories. For example, when asked about ethnicity, 53.3% (169/317; 95% CI 45.5-61) of the respondents in the NoSH group were extremely likely to share that type of data, compared to 45.17% (654/1448; 95% CI 41.3-49.1) in the SH group (*χ*^2^_4_=9.1; *P*=.06).

When we further categorized the SH group by whether individuals had contacted health services following SH (Multimedia Appendix 4), we found significant group differences for sharing data regarding marital status (*χ*^2^_4_=10.1; *P*=.04), mental (*χ*^2^_4_=31.2; *P*<.001), and physical health (*χ*^2^_4_=15.6; *P*=.004). Respondents who contacted health services were more likely to share mental health and physical health data, 34.3% (95% CI 28.0-41.2; 209/609) of those who had contacted services responded that they were “Extremely likely” to share mental health data compared with 21.6% (95% CI 15.8-28.7; 169/782) of those who had not contacted services. Similarly when asked about physical health data, 19.2% (95% CI 12.7-27.8; 117/609) of those who had contacted services for SH responded that they would be “extremely likely” to share this data compared with 12.3% (95% CI 6.7-20.9; 96/782) of those who had not contacted services.

The respondents were asked in a free text response “How do you feel about your social media posts being used for research? Would the data being anonymised make you feel differently?” A total of 39.49% (697/1765) of the respondents responded negatively, with 33.03% (583/1765) stating that they would only be happy to share this information if certain conditions were met (no significant difference between the SH and NoSH groups). Concerns were most commonly related to privacy and anonymity. Social media was often considered very personal information, and for some, it was more personal than medical data. Individuals who were positive about their data being used were under the assumption that their data were not private, with a respondent stating the following:

I make my social media accounts public by choice, therefore I wouldn’t mind my posts being used for the purpose of research–the social media companies themselves already do this whether you like it or not for advertising etc.

participant F51

This is in contrast with the views of those who responded negatively:

Social media should only be used in research if the person is completely happy and aware with where their data is taken. Pictures or videos should never be taken. If social media is used, then I feel it is no longer anonymised. People can look up specific captions and hashtags taken from posts, and you have lost complete anonymity.

participant F38

Respondents were skeptical about how it is possible to anonymize social media posts with concerns raised around images:

Most of the stuff I post is pictures therefore I’m not sure how that can be anonymised.

participant F715

Uncomfortable due to the lack of anonymity as it’s my face, you can’t really make that anonymous.

participant M51

Distinctions were made between different social media platforms and privacy settings:

I’d hate to have my social media posts used for research as it’s private for a reason. If it was anonymous, I’d feel better but only for things like twitter where there’s mostly just tweets rather than photos.

participant F412

#### Topic 2: Use of Mental Health Data

**Figure 2:**
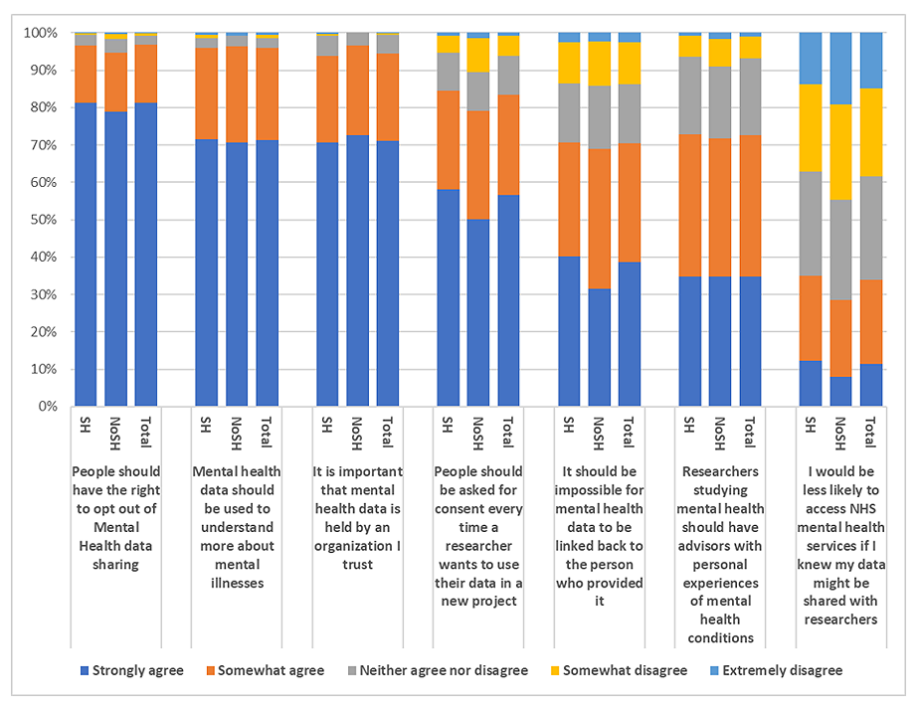
Responses to the question “When thinking about sharing mental health data, to what extent do you disagree or agree with the following statements?” NoSH: no history of self-harm; SH: history of self-harm.

Responses to the question “When thinking about sharing mental health data, to what extent do you disagree or agree with the following statements?” are shown in Figure 2 (Multimedia Appendix 5). Most respondents (1357/1765, 76.88%; 95% CI 74.5-79.1) strongly agreed that they should have the right to opt out of mental health data sharing, it is important that mental health data are held by an organization they trust (1188/1765, 67.31%; 95% CI 64.5-70.0), and mental health data should be used to understand more about mental illness (1203/1765, 68.16%; 95% CI 65.4-70.8). When asked whether they would be less likely to access NHS mental health services if they knew that their data would be shared with researchers just 10.9% (95% CI 7.0-16.4; 192/1671) responded “strongly agree.” Those who had contacted health services following SH were more likely to agree to the sharing of their data than those who had not contacted services (contacted—strongly disagree: 97/609, 15.9%; CI 9.6-25.1; contacted—somewhat disagree: 146/609, 24%; 95% CI 17.5-31.9; not contacted—strongly disagree: 84/782, 10.7%; 95% CI 5.3-19.9; not contacted—somewhat disagree: 162/782, 20.7%; 95% CI 14.9-27.9; *χ*^2^_4_= 12.5; *P*=.01). However, 10.88% (192/1765; 95% CI 7.0-16.4) of all the respondents strongly agreed that they would be less likely to access NHS mental health services if they knew that their data would be shared with researchers, with those with SH slightly more likely to agree than those without (Multimedia Appendix 6).

Those who contacted health services were more likely to agree that researchers studying mental health should have advisers with personal experiences of mental health conditions (contacted: 227/609, 37.3%; 95% CI 31.0-44.0; not contacted: 234/782, 29.9%; 95% CI 24.2-36.3; responded “strongly agree”; *χ*^2^_4_=12.5; *P*=.01). The SH and NoSH groups were comparable on most statements (*P* values=.35-.56). While there were some significant differences between groups, the NoSH group was small, and as such CIs were wide (Multimedia Appendix 5).

#### Topic 3: Factors Influencing Feelings Toward Use of Data

**Figure 3:**
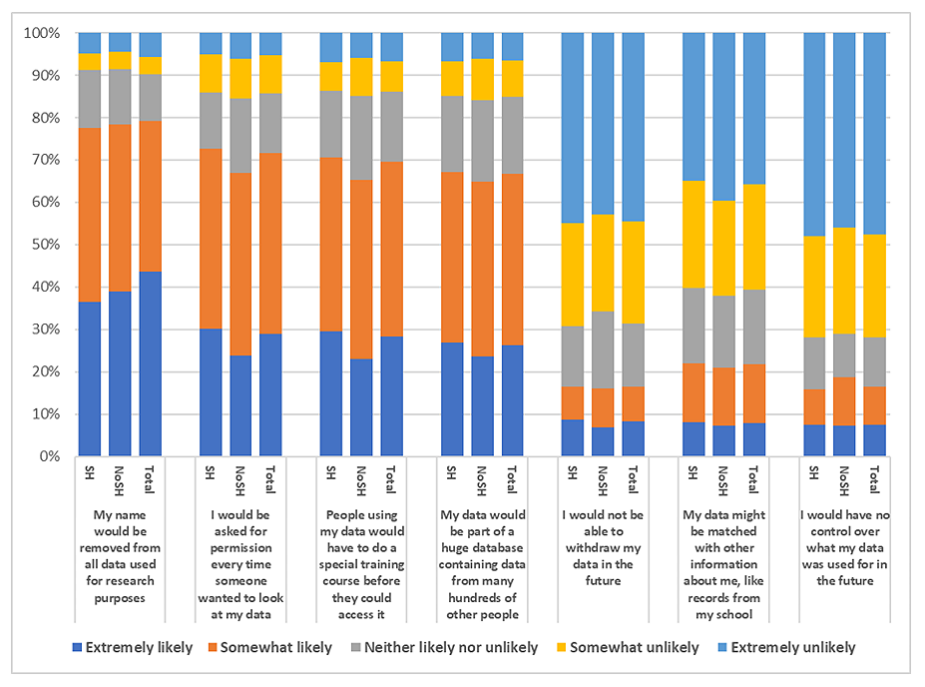
Responses to the question “How would the following measures change the likelihood that you would be willing to share your mental health data for research purposes?” NoSH: no history of self-harm; SH: history of self-harm.

Responses to the question “How would the following measures change the likelihood you would be willing to share your mental health data for research purposes?” are shown in Figure 3 (the distribution of answers is provided in Multimedia Appendix 7). Respondents were more likely to share data if names were removed from research data (extremely likely: 736/1765, 41.7%; 95% CI 38.1-45.4; somewhat likely: 599/1765, 33.94%; 95% CI 30.2-37.9), and permissions were asked every time the data were used (extremely likely: 496/1765, 28.1%; 95% CI 24.2-32.3; somewhat likely: 728/1765, 41.25%; 95% CI 37.7-44.9). In contrast, respondents were extremely unlikely to share data if they had no control over what their data were used for in the future (802/1765, 45.44%; 95% CI 42.0-49.0), would not be able to withdraw their data in the future (744/1765, 42.15%; 95% CI 38.6-45.8), or have their data matched with other data (603/1765, 34.16%; 95% CI 30.4-38.1). Comparison between the SH and NoSH groups revealed no statistical differences in the distributions of responses (*P*=.09-.72) nor were there any differences when comparing individuals who had or had not contacted health services following SH (*P*=.07-.80; Multimedia Appendix 8).

Analysis of free text responses suggested that young people would feel more positive about sharing their data if they were better informed about the nature of the research and potential benefits of services. They were asked the question “Would you like a better explanation of how your data is used and shared for research?” A total of 53.37% (942/1765) of the respondents responded positively or yes, with explanations required to be short and easy to understand. A common theme across all respondents (positively or yes and negatively or no) was wanting to know more about the research and its impact. For example, one respondent who stated that they would like a better explanation stated the following:

Yea, it would be good to know how this research will help people in the future who struggle with mental health problems.

participant F598

While another respondent who did not want any further explanation on how their data were used stated the following:

To be honest, as long as my personal info is not given away, I am not fussed. However, I think that anyone who took part should be allowed to view the paper in full once drafted or published. Normally you have to pay a fee to read a research paper, but this seems a bit unfair if you were part of the experiment!

participant F421

#### Topic 4: Trustworthiness of Organizations

**Figure 4:**
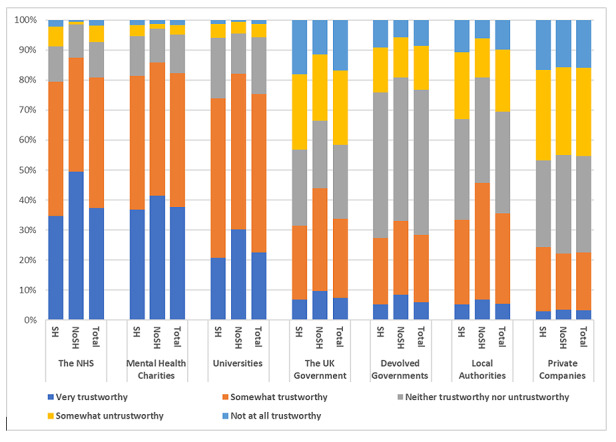
Responses to the question “In your opinion, how trustworthy are the following organizations when it comes to storing and using mental health data for research?” NoSH: no history of self-harm; NHS: National Health Service; SH: history of self-harm.

Respondents were presented with a list of organizations and asked, “In your opinion how trustworthy are the following organizations when it comes to storing and using mental health data for research?” (refer to Figure 4 and Multimedia Appendix 9 for details). Most respondents reported that mental health charities were the most trustworthy (very trustworthy: 651/1765, 36.88%; 95% CI 33.2-40.7; somewhat trustworthy: 767/1765, 43.46%; 95% CI 39.9-47.1) followed by the NHS (very trustworthy: 649/1765, 36.77%; 95% CI 33.1-40.6; somewhat trustworthy: 754/1765, 42.72%; 95% CI 39.2-46.3) and universities (very trustworthy: 383/1765, 21.7%; 95% CI 17.7-26.2; somewhat trustworthy: 900/1765, 50.99%; 95% CI 47.7-54.3). Private companies, local authorities, and devolved governments were rated as the least trustworthy organizations for storing and using mental health data. We observed significant differences in distributions of the degree of trustworthiness between the 2 groups for the NHS (*χ*^2^_4_=35.3; *P*<.001), universities (*χ*^2^_4_=17.5; *P*=.002), UK government (*χ*^2^_4_=19.7; *P*=.001), and local authorities (*χ*^2^_4_=27.0; *P*<.001). The NoSH group showed slightly higher trust in NHS, universities, and UK government in comparison to the SH group. For example, 48.9% (155/317; 95% CI 40.8-57.0) respondents of the NoSH group and 34.12% (494/1448; 95% CI 30.0-38.5) respondents of the SH group said that the NHS was “very trustworthy,” and 29.7% (94/317; 95% CI 20.9-40.1) respondents of the NoSH group and 19.96% (289/1448; 95% CI 15.6-25.1) respondents of the SH group said that universities were “very trustworthy.” We found similar distributions in the degree of trustworthiness related to all organizations between respondents with a history of SH independent of contact with health services (*P*=.08-.90; Multimedia Appendix 10).

The participants were asked the open-ended question, “Do you feel differently about research done by universities than that done by companies?” A total of 56.09% (990/1765) of the respondents responded that they felt more positively about research done by universities than companies (no significant differences between the SH and NoSH groups).

Positive responses were most frequently related to universities being regarded as more trustworthy, with educational institutions and the role of students being regarded positively:

Yes, as people from universities are younger and are more likely to understand.

participant F56

Universities were perceived to have better data security, and research was commonly described as feeling safer and more ethical with a focus on the greater good of developing the field and helping others:

Yes, if university research is being done, then it is often for the greater good but often with companies, I feel that our data can be misused.

participant F899

Around a third of respondents answered either positively, negatively, or conditionally to the question “How about information from companies such as Amazon, Google, or Fitbit being used for research?” Among positive responses were statements that these data were less invasive and personal than other data types. The most common negative responses were around data security and privacy:

I think the data these companies collect is wrong as I feel it violates our online privacy and kind of scares me to know they track your every action online.

participant M52

Consent and ethical issues were common among negative responses, as was the discussion of the use of data for profit:

No. Those purposes would be advertising purposes and that isn’t helping the public, only themselves at the expense of the person whom the data comes from.

participant F22

If the person is not aware that research is being done in them then I don’t think it is the right thing to do.

participant F1001

Responses appeared dependent on whether these types of data were considered relevant to mental health research. Fitbit was singled out as a company that individuals felt more comfortable with. The most frequent conditional responses were related to data being appropriately anonymized, having control over what data are shared (eg, exercise and sleep data from Fitbit but not location), and being given the option to consent. The use of data for the “greater good” also frequently appeared in conditional responses. A participant stated the following:

Only if it benefits other people and provides them help.

participant M87

#### Topic 5: The Use of Anonymized Health Care Data

Respondents were asked, “How would you feel about researchers linking things like your answers to questionnaires with anonymised healthcare data, and why?” A total of 91.9% (1622/1765) of the respondents answered, “I would be OK with this” and 745 (42.21%) individuals gave a free text response to the question “Why?” Of those who answered “I would be OK with this,” 52.1% (388/745) gave reasons related to the use of these data to help people:

If it helps other people and saves lives of people in healthcare crisis then it is a great thing to do. And if it has any chance of waking the government up to the fact that the older generation is not the only one that matters and that we desperately need help and that the world has changed and they have to change too, then of course I want to help.

participant F98

Of those who answered “I would not be OK with this,” the most frequent free text responses were related to data security, followed by ethical issues particularly related to consent:

I’m not wholly against my data anonymously being used for research, but I wouldn’t be happy if this was done without my knowledge/consent. It would feel like a violation and breach of trust between me and the healthcare professionals I had disclosed information to originally. It makes me feel like I’m just a set of data to scientists/researchers/doctors.

participant F225

Participants were asked to provide a free text response to the question “How do you feel about anonymous healthcare data being used for research?” Responses indicated there were no significant differences between the 2 groups. Across both groups, 66.4% (1172/1765) of the respondents stated that they felt positive about these data being used, with an additional 21.13% (373/1765) of the respondents stating that they would feel more positively about this if certain conditions were met, with data security and privacy (385/1765, 21.81%) and the way data are used (351/1765, 19.89%) standing out as the most prominent conditions. The most common negative responses fell under “features of research.” These were mostly related to concerns over how useful these kinds of data could be without the context or surrounding circumstances. This was followed by concerns over data security and privacy. The most common reason for a positive response was the use of data for “the greater good”:

I think it’s amazing because it might actually cause a big improvement in schools and how mental health is talked about, treated, and supported.

participant F289

The use of data for the “greater good” or for the purpose of helping others was also a common theme of conditional responses. Conditional responses were most frequently related to concerns over data security followed by ethical issues. A participant stated the following:

I think using this information is very beneficial, but people should be made more aware that their data could potentially be used and given an option to opt-out if they do not feel comfortable with this.

participant F74

While most respondents (1622/1765, 91.9%) were comfortable with questionnaire responses being linked with routinely collected health care data, some were not comfortable providing the data required for linkage. Participants were asked, “How would you feel about giving personal information such as address and date of birth for linking research and routine healthcare data?” A total of 24.14% (426/1765) of the individuals were happy to give both address and date of birth; however, 30.25% (534/1765) of the respondents stated that they would not be willing to provide either one. A further 18.53% (327/1765) of the respondents indicated that they may be more willing to give this information if certain conditions were met. No differences between the 2 groups were identified.

Individuals were more comfortable giving their date of birth than their address as this was thought to be less identifiable information, and 3.68% (65/1765) of the respondents were willing to give a generic location or postcode rather than a specific address. Other suggestions included the use of an NHS number. The most common reasons for not wanting to give this information were concerns related to data security, including the security of giving this information online. Others were concerned about other people that they live with, being written to at their home address and the impact of being identified:

Date of birth fine. Address no. I just know my mum wouldn’t be ok with it.

participant F301

I would be scared. What if i told a researcher about my self-harm issues and I get sectioned again?

participant F149

Those who were willing to share the required information were most likely to give the “greater good” as a reason and a willingness to contribute data to help others.

## Discussion

### Principal Findings

We examined young people’s opinions on the use of their routinely collected data for mental health research using a web-based survey. We evaluated any differences in the views of young people with or without a history of SH and those in contact with services for SH. Views on the use of mental health data for research were mostly positive with most emphasizing the importance of the benefit to the public and patients. Respondents were less likely to want to share their social media data, which they considered to be more personal compared to their health care data. Respondents stressed the importance of anonymity and the need for an appropriate ethical framework. Individuals who reported a history of SH and subsequently contacted health services were more willing to share mental and physical health data compared with those who had not contacted services. We did not observe any significant differences between the SH and NoSH groups.

### Use of Data for Research

The use of data for mental health research of public and patient benefit was viewed positively across all questions asked. Young people were less likely to share their social media and financial data than ethnicity, marital status, and mental and physical health data for research.

Individuals both with and without a history of SH reported broadly similar views regarding their data being used; however, there were clear indications across responses that the importance of data sharing and its use for mental health research for young people and their attitudes toward it was associated with their characteristics and experience. Responses indicated that those with a history of SH might be less likely to use NHS mental health services if they knew that their data would be shared with researchers (although numbers were small); however, this was reversed in those with a history of SH but contacted services. Individuals who reported SH and contacted health services were more willing to share mental and physical health data compared with those who had not. This is further supported in the work done by Kirkham et al [12] with NHS users and Watson et al [13] indicating that while there were concerns that data misuse might worsen stigma, attitudes were largely positive. Favorable experiences of mental health services were likely to result in a higher willingness to share mental health data.

### Influencing Factors on the Use of Data

In keeping with older generations [1,6-8], young people share a mostly positive attitude toward their health care data being linked and shared for research purposes, with many relating their positive opinions with the use of this data for public and patient benefit. Respondents stressed the importance of anonymity and the need for appropriate ethical frameworks in keeping with previous work emphasizing the importance of identity protection, data security, transparency, and safeguarding policies while ensuring the use of data is within the public’s best interest [4,5].

Young people were less willing to share their social media data and considered this more personal than health care data. While anonymity was consistently reported as important, respondents varied in their understanding of how their social media data could be used and anonymized, particularly images. This is supported by existing literature in relation to young people’s lack of awareness and understanding of privacy issues online [31,33]. This contradicts the concept that young people do not care about privacy simply because they are heavily and publicly engaged in the online world [45]. Views on the use of social media data were shared by an adult population in a qualitative study performed by Beninger [46], where users expressed diverse responses toward social media research, ranging from skepticism on how it could be used for research to acceptance and ambivalence that stemmed from the idea that social media is already public and as such may inevitably be used. While some views were similar across generations, adults were less reluctant to share their social media content, as long as conditions, such as privacy and consent were met [47].

Respondents expressed negative feelings about the use of their data for profit or advertising, and this was frequently referenced in relation to the use of their data by private companies.

Anonymity, consent, and research skills were key factors that influenced positive feelings toward sharing mental health data. Losing control over their data, linking with other datasets, and inability to withdraw from data sharing were concerns that influenced negative feelings and unwillingness to share data. Another common theme signified respondents’ willingness to share their mental health data only if other conditions were met, such as the ability to opt out of data sharing and if data are used to understand more about mental illness. Respondents appreciated the importance of mental health research to better understand mental health and inform services, especially when given more control over their data.

### Trust in Organizations

Most respondents strongly agreed that they should have the right to opt out of mental health data sharing and that it is important that mental health data are held by an organization they trust with charities, the NHS, and universities being the most trusted and private companies and industry the least. This aligns with views from the adult population showing strong support for the NHS and academic institutions for data sharing [16,48]. Only one-tenth strongly agreed with the statement that they would be less likely to access NHS mental health services if they knew that their data would be shared with researchers.

### Strengths and Limitations

This is a large survey with >1700 respondents and represents the first of its kind with young people aged 16 to 24 years with a history of SH. It is also the first to compare the views of those who have contacted health services for SH and those who have not. The combination of fixed- and open-response questions provides a richness of data and a wider scale of insights that can be used to guide future research. Using a convenience sample allowed a rapid low-cost engagement with young people. However, convenience samples are inherently biased and not representative, and results must be viewed with a level of caution [49]. The sample of respondents primarily consisted of younger women and girls, and the creative used to recruit respondents held a message of “mental health data and research,” which is likely to be the reason for a large proportion of people with a history of SH. The small number of participants in the age group of 18 to 24 years as well as the small number of men and boys, meant that a meaningful breakdown by age and gender was not possible. Online survey respondents are often younger, well-educated, and have English as their first language [50,51].

Online surveys may be subject to fraudulent responses, a limited ability to target certain features in the sample needed, and may be limited by the absence of an interviewer to clarify questions [52]. Some of these limitations were mitigated by ensuring the visual material incorporated a clear message to target respondents and designing the survey questions to be simple and easy to comprehend. The use of social media to recruit respondents was highly effective in terms of recruitment, cost, and time when compared with traditional recruitment approaches. It also facilitated the participation of underserved groups, who may face stigma or other barriers to taking part in research [53,54]. People with mental health issues are more likely to fill out mental health–related questionnaires than the remainder of the population, with this difference amplified for online surveys. This does not present an issue for research looking to gather opinions of individuals with mental health issues but is problematic for prevalence research [50]. Individuals with a history of SH were purposefully sampled in this study, and, notably, there was high engagement from this group. This was likely related to the nature of the social media campaign, which used visual materials, such as videos and posters, and was conducted largely over Instagram.

### Implications for Policy and Practice

Our findings, particularly patterns based on respondents’ characteristics and experiences, highlight the importance of involving young people from diverse backgrounds at all stages of research development. Those with a history of SH valued participation of those with lived experience in mental health data research more highly than those without a history of SH. They also had reduced levels of trust in the use of their data for research across all organizations compared to those without. There is work to be done to increase confidence in individuals with a history of SH. We need to develop a shared understanding of consent, the “five safes framework” [55], anonymization processes, and the public benefits of mental health data science. This could be supported by coproduced resources and platforms for discussion, for example, online or in educational settings. Carter et al [56] stressed the importance of public engagement on a social level, rather than simply focusing on the requirements of formal regulations, in order to facilitate understanding and trust from the public on the topic of sharing their health data. Respondents repeatedly stated that they would like to know about the research being done. One of the key factors influencing positive opinions toward the use of data for research was the desire for it to be used to help others. Raising awareness of the ways in which research can be used to improve services and lives would likely have a positive impact on attitudes toward data sharing. Any campaigns to raise awareness should be widely accessible to young people with social media likely to be a useful way to reach them. Accessible case studies, demonstrating the impact of research on peoples’ lives, lay research summaries, and real-world impacts on services should be widely disseminated beyond academic audiences. Research findings should be made accessible to individuals who have participated, in ways that are suitable for their age or background.

There appears to be a greater need to educate young people about how their social media data are used and their data availability for research on platforms as part of the terms of service. Standardized anonymization processes such as those suggested by the British Psychological Society [20] would represent a positive step toward clarifying how identities are protected when using these data for research. Procedures should include consideration of the republishing of photos and direct quotes in research outputs, as even with usernames or personal details removed, there is still the potential for these to be traced back to the individual [22,57]. Resources demonstrating how images can be labeled, processed, and analyzed without a researcher accessing them directly should be developed. Special consideration should be given to communicating with vulnerable groups including children and adolescents and people with mental health issues [22].

Future research could explore the acceptability of data use from a wider range of services such as schools, universities, or social care. In addition, recruitment to increase the number of boys or men and young adults would provide a more comprehensive overview of the opinions of this group.

### Conclusions

This is the first survey to gather in-depth opinions on the use of big data for mental health research from young people, specifically including those with a history of SH. Young people are most definitely aware, and indeed they care about how their data are being used and for what purposes. They are largely positive about the use of health care data (mental or physical) for research and generally echo the opinions of older age groups raising issues around data security and the use of data for public interest. In addition, this study demonstrates that young people feel strongly about the use of their social media data for research, which was felt to be more personal. Those who had been in contact with health care services for SH were more positive about these data being used to improve services. There is a need for greater awareness of how different sources of data are used for research with a focus on anonymization, privacy, and the way in which data are used to help others.

## Appendix

Multimedia Appendix 1 Survey questions.

Multimedia Appendix 2 Summary of chi-square statistics for cross-tabulations on response rates of closed-end questions stratified by gender, age, history of self-harm, and contact with health services following self-harm.

Multimedia Appendix 3 Distribution of answers to the question “Thinking about data more generally (not only health data), how likely would you be to share the following types of data for research purposes,” stratified by history of self-harm and no history of self-harm groups.

Multimedia Appendix 4 Distribution of answers to the question “Thinking about data more generally (not only health data), how likely would you be to share the following types of data for research purposes?” from the history of self-harm group, stratified by having contact with health services following self-harm.

Multimedia Appendix 5 Distribution of answers to the question “When thinking about sharing mental health data, to what extent do you disagree or agree with the following statements?” stratified by history of self-harm and no history of self-harm groups.

Multimedia Appendix 6 Distribution of answers to the question “When thinking about sharing mental health data, to what extent do you disagree or agree with the following statements?” from the history of self-harm group stratified by having contact with health services following self-harm.

Multimedia Appendix 7 Distribution of answers to the question “How would the following measures change the likelihood that you would be willing to share your mental health data for research purposes,” stratified by history of self-harm and no history of self-harm groups.

Multimedia Appendix 8 Distribution of answers to the question “How would the following measures change the likelihood that you would be willing to share your mental health data for research purposes” from the history of self-harm group stratified by having contact with health services following self-harm.

Multimedia Appendix 9 Distribution of answers to the question “In your opinion, how trustworthy are the following organisations when it comes to storing and using mental health data for research?” stratified by history of self-harm and no history of self-harm groups.

Multimedia Appendix 10 Distribution of answers to the question ‘‘In your opinion, how trustworthy are the following organisations when it comes to storing and using mental health data for research?” from the history of self-harm group stratified by having contact with health services following self-harm.

## Abbreviations

GP: general practitioner
NHS: National Health Service
NoSH: no history of self-harm
SH: self-harm
