# Supplementary material for: Exploring the Views of Young People, Including Those With a History of Self-Harm, on the Use of Their Routinely Generated Data for Mental Health Research: Web-Based Cross-Sectional Survey Study
Source: JMIR Ment Health. 2025 Mar 12;12:e60649. doi: 10.2196/60649 (PMC11947630; doi:10.2196/60649)
Supplement: Multimedia Appendix 1 [file mental_v12i1e60649_app1.docx]

**Multimedia Appendix 1: Survey Questions**

**Instructions for participants**

Below are a series of questions looking to learn more about you and your views on how we can improve research and better push for change.

There are no right or wrong answers and you don’t need to worry about spelling or grammar – what is really important are your stories and views.

There is no recommended length for answers and you are free to give as much or as little detail as you like. The more you can tell us the better and if you feel able to give detailed answers, this is great as it will give us even more to work with.

All questions are optional and if you do not want to answer a particular question then just leave it blank.

**Topic area 1: A bit about yourself**

Age: Gender: Location: England Wales Scotland NI

1. **Have you ever done any of the following intending to harm yourself (tick all that apply):** Self-injury such as self-cutting, scratching or hitting etc.

Ingesting medication in excess of the normal dose

Swallowing dangerous objects or products

Stopping prescribed medication

Something not listed

Prefer not answer

1. **Have you harmed yourself with the intention to end your life**

Yes No Prefer not to answer

1. **Was this in the last 12 months**

Yes No Prefer not to answer

1. **Following any time when you took an overdose or intentionally tried to harm yourself did you (tick all that apply)**

Need hospital treatment (e.g. A&E)

See anyone from psychiatric or mental health services, including liaison services

See your GP

Receive help from friends/family/neighbours

Use a helpline/voluntary organization

None of the above

Prefer not to answer

**Topic area 2: Your views on research related to mental health or self-harm [free text]**

1. What kind of things do you think research should focus on? E.g. the role of the community, schools or the internet [free text response]
2. What kinds of things would put you off taking part in research? [free text response]
3. We are hoping to learn more about the way people prefer to take part in research. Would you prefer to take part in research

Online

In person

By phone

Other/comments [Space for free text]

1. How would you prefer to answer questions?

Check box

Space to write

Other/comments [Space for free text]

1. Have you ever taken part in research related to self-harm or mental health before? If so could you tell us a bit more about it? What did you or like/dislike

[Free text response]

1. Was there anything you would have liked to ask the researcher but didn’t get a chance to?

[Free text response]

**Topic area 3: Big data and your data for research**

We want to know more about your feelings on the use of your data for research. We are interested in your feelings about healthcare data and also other personal data such as social media posts. This is only to find out your views. We are not using any other data in this project.

As patients whenever we have contact with GPs or hospitals, data is collected as part of that visit. This data can be anonymised and used for research to help make the health service work better for you and others.

Some projects link things like questionnaire answers from people with this anonymised healthcare information. This can help answer questions like whether watching lots of TV is bad for your health or whether people with certain types of jobs spend more or less time in hospital. This information is looked after very carefully and this type of research is anonymous, held securely and privacy protected.

1. How do you feel about anonymous healthcare data being used for research? [Free text response]
2. Would you like a better explanation of how your data is used and shared for research? [Free text response]
3. How would you feel about researchers linking things like your answers to questionnaires with anonymised healthcare data?

I would be ok with this

I would not be ok with this

Why? [Space for free text]

1. In order to link between research and routine healthcare data some personal details are needed including address and date of birth. These get sent to NHS information partners to make the link. Researchers never see this information and it is destroyed after the link is made. How would you feel about giving personal information such as address and date of birth for this purpose? (You do not have to give this here) [Free text response]
2. How do you feel about your social media posts being used for research? Would the data being anonymised make you feel differently? [Free text response]
3. How about information from companies such as Amazon, Google or Fitbit being used for research? [Free text response]
4. Do you feel differently about research done by universities than that done by companies?
5. Thinking about data more generally (not only health data), how likely would you be to share the following types of data for research purposes

|  | Extremely unlikely | Somewhat unlikely | Neither likely nor unlikely | Somewhat likely | Extremely likely |
| --- | --- | --- | --- | --- | --- |
| Social media posts |  |  |  |  |  |
| Physical health data |  |  |  |  |  |
| Financial information (e.g. Credit rating) |  |  |  |  |  |
| Mental health data |  |  |  |  |  |
| Employment history |  |  |  |  |  |
| Ethnicity |  |  |  |  |  |
| Marital status |  |  |  |  |  |

1. In your opinion, how trustworthy are the following organisations when it comes to storing and using mental health data for research

|  | Not at all trustworthy |  |  |  | Very trustworthy |
| --- | --- | --- | --- | --- | --- |
| The NHS |  |  |  |  |  |
| The UK government |  |  |  |  |  |
| Your local authority/council |  |  |  |  |  |
| Universities |  |  |  |  |  |
| Mental health charities |  |  |  |  |  |
| Private companies |  |  |  |  |  |
| Devolved governments (e.g. Scottish, Welsh or Northern Irish governments) |  |  |  |  |  |

1. How would the following measures change the likelihood that you would be willing to share your mental health data for research purposes

|  | Extremely unlikely | Somewhat unlikely | Neither likely nor unlikely | Somewhat likely | Extremely likely |
| --- | --- | --- | --- | --- | --- |
| I would be asked for permission every time someone wanted to look at my data |  |  |  |  |  |
| My data would be part of a huge database containing data from many hundreds of other people |  |  |  |  |  |
| I would have no control over what my data was used for in the future |  |  |  |  |  |
| My name would be removed from all data used for research purposes |  |  |  |  |  |
| My data might be matched with other information about me, like records from my school |  |  |  |  |  |
| People using my data would have to do a special training course before they could access it |  |  |  |  |  |
| I would not be able to withdraw my data in the future |  |  |  |  |  |

1. When thinking about sharing mental health data, to what extent do you disagree or agree with the following statements?

|  | Strongly disagree | Somewhat disagree | Neither agree nor disagree | Somewhat agree | Strongly agree |
| --- | --- | --- | --- | --- | --- |
| Mental health data should be used to understand more about mental illnesses |  |  |  |  |  |
| It should be impossible for mental health data to be linked back to the person who provided it |  |  |  |  |  |
| People should be asked for consent every time a researcher wants to use their data in a new project |  |  |  |  |  |
| People should have the right to opt out of mental health data sharing |  |  |  |  |  |
| I would be less likely to access NHS mental health services if I knew my data might be shared with researchers |  |  |  |  |  |
| It is important that mental health data is held by an organisation I trust |  |  |  |  |  |
| Researchers studying mental health should have advisors with personal experiences of mental health conditions |  |  |  |  |  |

1. Do you have any other thoughts or comments about how personal/healthcare data is used? [Free text response]

**Topic area 4: Things researchers should know**

1. Please use this space to tell us anything you think researchers should know. This can be anything you would like to tell us or that you feel could help research going forward

[Free text response]

1. Do you have any other thoughts, comments or suggestions?

[Free text response]
