# Supplementary material for: Exploring the Views of Young People, Including Those With a History of Self-Harm, on the Use of Their Routinely Generated Data for Mental Health Research: Web-Based Cross-Sectional Survey Study
Source: JMIR Ment Health. 2025 Mar 12;12:e60649. doi: 10.2196/60649 (PMC11947630; doi:10.2196/60649)
Supplement: Multimedia Appendix 2 [file mental_v12i1e60649_app2.docx]

Supplementary Table 1 Summary of chi-square statistics for cross-tabulations on response rates of closed-end questions stratified by gender, age, history of self-harm and contact to health services following self-harm

|  |  | **Gender** | | |  | **Age** | | |  | **History of self-harm** | | |  | **Contact to health services following self-harm** | | |
| --- | --- | --- | --- | --- | --- | --- | --- | --- | --- | --- | --- | --- | --- | --- | --- | --- |
| **Question** | **Item** | **chi-square** | **df** | **p-value** |  | **chi-square** | **df** | **p-value** |  | **chi-square** | **df** | **p-value** |  | **chi-square** | **df** | **p-value** |
| How would you feel about researchers linking things like your answers to questionnaires with anonymised healthcare data? | - | 11.2 | 2 | .004 |  | 17.6 | 4 | .002 |  | 63.4 | 1 | <.001 |  | 16.5 | 1 | <.001 |
|  |  |  |  |  |  |  |  |  |  |  |  |  |  |  |  |  |
| Thinking about data more generally (not only health data), how likely would you be to share the following types of data for research purposes? | Ethnicity | 14.9 | 2 | <.001 |  | 20.2 | 4 | <.001 |  | 62.6 | 1 | <.001 |  | 10.1 | 1 | .002 |
|  | Marital status | 15.0 | 2 | <.001 |  | 19.0 | 4 | <.001 |  | 63.6 | 1 | <.001 |  | 10.2 | 1 | .001 |
|  | Mental health data | 14.9 | 2 | <.001 |  | 19.6 | 4 | <.001 |  | 64.4 | 1 | <.001 |  | 9.9 | 1 | .002 |
|  | Physical health data | 15.0 | 2 | <.001 |  | 19.7 | 4 | <.001 |  | 64.1 | 1 | <.001 |  | 10.1 | 1 | .002 |
|  | Employment history | 14.9 | 2 | <.001 |  | 19.3 | 4 | <.001 |  | 62.3 | 1 | <.001 |  | 9.8 | 1 | .002 |
|  | Social media posts | 14.5 | 2 | <.001 |  | 18.5 | 4 | <.001 |  | 66.3 | 1 | <.001 |  | 11.0 | 1 | <.001 |
|  | Financial information | 14.6 | 2 | <.001 |  | 18.3 | 4 | .001 |  | 64.2 | 1 | <.001 |  | 10.2 | 1 | .001 |
|  |  |  |  |  |  |  |  |  |  |  |  |  |  |  |  |  |
| When thinking about sharing mental health data, to what extent do you disagree or agree with the following statements? | People should have the right to opt out of mental health data sharing | 16.7 | 2 | <.001 |  | 24.9 | 4 | <.001 |  | 59.8 | 1 | <.001 |  | 12.0 | 1 | <.001 |
|  | Mental health data is held by an organisation I trust | 17.1 | 2 | <.001 |  | 25.7 | 4 | <.001 |  | 63.2 | 1 | <.001 |  | 12.2 | 1 | <.001 |
|  | Mental health data should be used to understand more about mental illnesses | 16.2 | 2 | <.001 |  | 24.8 | 4 | <.001 |  | 63.3 | 1 | <.001 |  | 11.6 | 1 | <.001 |
|  | People should be asked for consent every time a researcher wants to use their data in a new project | 16.7 | 2 | <.001 |  | 23.9 | 4 | <.001 |  | 61.0 | 1 | <.001 |  | 11.0 | 1 | <.001 |
|  | Researchers should have advisors with personal experiences of MH | 16.2 | 2 | <.001 |  | 24.6 | 4 | <.001 |  | 62.6 | 1 | <.001 |  | 12.8 | 1 | <.001 |
|  | Impossible for mental health data to be linked back to the person who provided it | 16.3 | 2 | <.001 |  | 25.0 | 4 | <.001 |  | 63.1 | 1 | <.001 |  | 11.4 | 1 | <.001 |
|  | Would be less likely to access NHS mental health services if I knew my data might be shared with researchers | 16.9 | 2 | <.001 |  | 24.6 | 4 | <.001 |  | 60.5 | 1 | <.001 |  | 12.3 | 1 | <.001 |
|  |  |  |  |  |  |  |  |  |  |  |  |  |  |  |  |  |
| How would the following measures change the likelihood that you would be willing to share your mental health data for research purposes? | My name would be removed from all data | 15.2 | 2 | <.001 |  | 22.8 | 4 | <.001 |  | 60.6 | 1 | <.001 |  | 10.9 | 1 | <.001 |
|  | Asked for permission every time someone wanted to look at my data | 13.8 | 2 | .001 |  | 23.1 | 4 | <.001 |  | 62.6 | 1 | <.001 |  | 11.0 | 1 | <.001 |
|  | People would have to do training course before data access | 15.9 | 2 | <.001 |  | 25.5 | 4 | <.001 |  | 59.3 | 1 | <.001 |  | 10.7 | 1 | .001 |
|  | My data would be part of a huge database containing data from other people | 14.3 | 2 | <.001 |  | 23.5 | 4 | <.001 |  | 60.4 | 1 | <.001 |  | 10.5 | 1 | .001 |
|  | I would not be able to withdraw my data in the future | 15.9 | 2 | <.001 |  | 24.3 | 4 | <.001 |  | 59.3 | 1 | <.001 |  | 10.2 | 1 | .001 |
|  | My data might be matched with other information about me | 15.0 | 2 | <.001 |  | 23.8 | 4 | <.001 |  | 60.3 | 1 | <.001 |  | 10.4 | 1 | .001 |
|  | I would have no control over what my data was used for in the future | 15.3 | 2 | <.001 |  | 23.5 | 4 | <.001 |  | 61.4 | 1 | <.001 |  | 11.3 | 1 | <.001 |
|  |  |  |  |  |  |  |  |  |  |  |  |  |  |  |  |  |
| In your opinion, how trustworthy are the following organisations when it comes to storing and using mental health data for research? | The NHS | 14.5 | 2 | <.001 |  | 21.1 | 4 | <.001 |  | 65.0 | 1 | <.001 |  | 12.5 | 1 | <.001 |
|  | Mental health charities | 15.4 | 2 | <.001 |  | 21.6 | 4 | <.001 |  | 65.2 | 1 | <.001 |  | 11.3 | 1 | <.001 |
|  | Universities | 13.9 | 2 | <.001 |  | 9.9 | 4 | .043 |  | 58.8 | 1 | <.001 |  | 7.2 | 1 | .007 |
|  | The UK government | 15.0 | 2 | <.001 |  | 20.7 | 4 | <.001 |  | 65.7 | 1 | <.001 |  | 13.2 | 1 | <.001 |
|  | Devolved governments | 16.8 | 2 | <.001 |  | 22.2 | 4 | <.001 |  | 60.9 | 1 | <.001 |  | 10.1 | 1 | .001 |
|  | Your local authority/council | 15.4 | 2 | <.001 |  | 18.3 | 4 | .001 |  | 65.2 | 1 | <.001 |  | 11.7 | 1 | <.001 |
|  | Private companies | 15.4 | 2 | <.001 |  | 21.4 | 4 | <.001 |  | 63.9 | 1 | <.001 |  | 11.1 | 1 | <.001 |
