# Supplementary material for: Exploring the Views of Young People, Including Those With a History of Self-Harm, on the Use of Their Routinely Generated Data for Mental Health Research: Web-Based Cross-Sectional Survey Study
Source: JMIR Ment Health. 2025 Mar 12;12:e60649. doi: 10.2196/60649 (PMC11947630; doi:10.2196/60649)
Supplement: Multimedia Appendix 3 [file mental_v12i1e60649_app3.docx]

Supplementary Table 2a Distribution of answers to the question ‘Thinking about data more generally (not only health data), how likely would you be to share the following types of data for research purposes’ stratified by SH^a^ and NoSH^b^ groups %(95% CI; n]^c^

|  |  | Extremely likely | Somewhat likely | Neither likely nor unlikely | Somewhat unlikely | Extremely unlikely |
| --- | --- | --- | --- | --- | --- | --- |
| Ethnicity | SH | 45.2(41.3-49.1; n=654) | 29.4(25.2-34.0; n=426) | 14.5(10.2-20.2; n=210) | 3.3(0.4-14.2; n=48) | 5.3(1.7-13.6; n=77) |
|  | NoSH | 53.3(45.5-61.0; n=169) | 25.6(16.8-36.7; n=81) | 11.7(4.0-27.4; n=37) | 4.7(0.1-31.6; n=15) | 3.8(0.0-34.9; n=12) |
|  | ALL | 46.6(43.2-50.1; n=823) | 28.7(24.9-32.9; n=507) | 14.0(10.0-19.1; n=247) | 3.6(0.7-12.5; n=63) | 5.0(1.8-12.5; n=89) |
| Marital status | SH | 39.0(35.0-43.2; n=565) | 25.3(21.0-30.2; n=367) | 20.9(16.5-26.0; n=302) | 4.9(1.4-13.6; n=71) | 7.4(3.5-14.5; n=107) |
|  | NoSH | 45.4(37.2-53.9; n=144) | 24.6(15.9-35.9; n=78) | 18.0(9.5-30.8; n=57) | 4.7(0.1-31.6; n=15) | 5.7(0.3-29.5; n=18) |
|  | ALL | 40.2(36.6-43.9; n=709) | 25.2(21.3-29.6; n=445) | 20.3(16.4-25.0; n=359) | 4.9(1.6-12.4; n=86) | 7.1(3.5-13.5; n=125) |
| Mental health data | SH | 26.9(22.6-31.6; n=389) | 47.9(44.2-51.7; n=694) | 10.7(6.5-16.9; n=155) | 7.7(3.7-14.7; n=111) | 5.0(1.5-13.6; n=72) |
|  | NoSH | 27.8(19.0-38.5; n=88) | 49.2(41.2-57.3; n=156) | 10.4(3.0-27.1; n=33) | 7.3(1.0-27.7; n=23) | 4.4(0.0-32.5; n=14) |
|  | ALL | 27(23.1-31.3; n=477) | 48.2(44.8-51.6; n=850) | 10.7(6.8-16.2; n=188) | 7.6(3.9-13.8; n=134) | 4.9(1.6-12.4; n=86) |
| Physical health data | SH | 15.1(10.8-20.7; n=219) | 44.5(40.7-48.5; n=645) | 17.5(13.1-22.8; n=253) | 12.6(8.4-18.5; n=183) | 8.3(4.3-15.1; n=120) |
|  | NoSH | 17.7(9.2-30.6; n=56) | 46.1(37.8-54.5; n=146) | 17.0(8.6-30.2; n=54) | 12.6(4.8-27.7; n=40) | 5.7(0.3-29.5; n=18) |
|  | ALL | 15.6(11.6-20.5; n=275) | 44.8(41.3-48.4; n=791) | 17.4(13.4-22.2; n=307) | 12.6(8.7-17.9; n=223) | 7.8(4.1-14.0; n=138) |
| Employment history | SH | 12.1(7.8-18.1; n=175) | 28.7(24.4-33.3; n=415) | 24.7(20.3-29.5; n=357) | 17.7(13.3-23.0; n=256) | 14.6(10.2-20.2; n=211) |
|  | NoSH | 16.4(8.0-29.8; n=52) | 33.1(24.4-43.1; n=105) | 21.5(12.8-33.4; n=68) | 16.1(7.8-29.6; n=51) | 12(4.3-27.5; n=38) |
|  | ALL | 12.9(8.9-18.1; n=227) | 29.5(25.6-33.6; n=520) | 24.1(20.1-28.5; n=425) | 17.4(13.4-22.2; n=307) | 14.1(10.2-19.2; n=249) |
| Social media posts | SH | 6.6(2.7-14.1; n=95) | 25.8(21.5-30.6; n=373) | 17.7(13.4-23.1; n=257) | 26.2(21.9-31.0; n=380) | 22.0(17.7-27.1; n=319) |
|  | NoSH | 7.3(1.0-27.7; n=23) | 24.9(16.2-36.1; n=79) | 13.9(5.8-28.3; n=44) | 27.8(19.0-38.5; n=88) | 24.9(16.2-36.1; n=79) |
|  | ALL | 6.7(3.1-13.2; n=118) | 25.6(21.7-29.9; n=452) | 17.1(13.1-21.9; n=301) | 26.5(22.6-30.8; n=468) | 22.5(18.6-27.0; n=398) |
| Financial information (e.g. Credit rating) | SH | 4.5(1.1-13.6; n=65) | 13.3(9.0-19.1; n=192) | 21.3(17.0-26.4; n=309) | 23.8(19.5-28.7; n=345) | 34.9(30.8-39.3; n=506) |
|  | NoSH | 4.4(0.0-32.5; n=14) | 16.1(7.8-29.6; n=51) | 19.9(11.3-32.2; n=63) | 27.4(18.7-38.2; n=87) | 30.9(22.2-41.2; n=98) |
|  | ALL | 4.5(1.3-12.4; n=79) | 13.8(9.8-18.9; n=243) | 21.1(17.1-25.6; n=372) | 24.5(20.5-28.9; n=432) | 34.2(30.5-38.2; n=604) |
| 1. Self-harm group 2. No self-harm group 3. No response =<5% | | | | | | |
