# Supplementary material for: Exploring the Views of Young People, Including Those With a History of Self-Harm, on the Use of Their Routinely Generated Data for Mental Health Research: Web-Based Cross-Sectional Survey Study
Source: JMIR Ment Health. 2025 Mar 12;12:e60649. doi: 10.2196/60649 (PMC11947630; doi:10.2196/60649)
Supplement: Multimedia Appendix 4 [file mental_v12i1e60649_app4.docx]

Supplementary Table 2b Distribution of answers to the question ‘Thinking about data more generally (not only health data), how likely would you be to share the following types of data for research purposes?’ from the SH group stratified by having contact to health services following self-harm %(95% CI; n)^a^

|  | Contact to health services^b^ | Extremely likely | Somewhat likely | Neither likely nor unlikely | Somewhat unlikely | Extremely unlikely |
| --- | --- | --- | --- | --- | --- | --- |
| Ethnicity | No | 43.4(38.0-48.8; n=339) | 31.3(25.7-37.6; n=245) | 16.1(10.4-24.0; n=126) | 2.8(0.0-22.5; n=22) | 4.9(0.8-18.5; n=38) |
|  | Yes | 47.9(42.1-53.8; n=292) | 26.4(20.0-34.1; n=161) | 12.8(6.6-22.8; n=78) | 4.1(0.2-22.5; n=25) | 5.9(1.1-20.5; n=36) |
| Marital status | No | 36.8(31.3-42.7; n=288) | 27.5(21.7-34.1; n=215) | 22.6(16.8-29.6; n=177) | 4.6(0.6-18.7; n=36) | 6.6(1.9-18.1; n=52) |
|  | Yes | 42.0(36.0-48.4; n=256) | 23.0(16.5-31.0; n=140) | 18.2(11.8-26.9; n=111) | 5.6(0.9-20.7; n=34) | 8.2(2.7-20.4; n=50) |
| Mental health data | No | 21.6(15.8-28.7; n=169) | 51.5(46.5-56.5; n=403) | 12.1(6.6-20.8; n=95) | 8.2(3.1-18.5; n=64) | 5.5(1.1-18.2; n=43) |
|  | Yes | 34.3(28.0-41.2; n=209) | 43.8(37.8-50.0; n=267) | 8.4(2.8-20.4; n=51) | 6.6(1.5-20.3; n=40) | 4.4(0.4-21.9; n=27) |
| Physical health data | No | 12.3(6.7-20.9; n=96) | 45.3(40.0-50.6; n=354) | 19.4(13.7-26.8; n=152) | 13.4(7.8-21.8; n=105) | 8.4(3.3-18.6; n=66) |
|  | Yes | 19.2(12.7-27.8; n=117) | 43.8(37.8-50.0; n=267) | 15.1(8.8-24.4; n=92) | 11.7(5.6-22.0; n=71) | 7.7(2.3-20.3; n=47) |
| Employment history | No | 10.5(5.1-19.7; n=82) | 27.7(22.0-34.3; n=217) | 26.6(20.8-33.2; n=208) | 18.9(13.1-26.4; n=148) | 14.7(9.0-22.8; n=115) |
|  | Yes | 14.6(8.3-24.1; n=89) | 28.9(22.5-36.3; n=176) | 22.2(15.7-30.3; n=135) | 16.9(10.5-25.9; n=103) | 14.4(8.2-23.9; n=88) |
| Social media posts | No | 5.5(1.1-18.2; n=43) | 25.6(19.8-32.3; n=200) | 19.6(13.8-26.9; n=153) | 25.4(19.7-32.2; n=199) | 22.8(17.0-29.8; n=178) |
|  | Yes | 8.0(2.6-20.3; n=49) | 25.8(19.3-33.5; n=157) | 15.8(9.4-24.9; n=96) | 27.9(21.5-35.4; n=170) | 20.5(14.0-28.9; n=125) |
| Financial information (e.g. Credit rating) | No | 4.0(0.3-19.5; n=31) | 13.0(7.5-21.5; n=102) | 20.1(14.3-27.4; n=157) | 25.8(20.1-32.5; n=202) | 35.7(30.1-41.6; n=279) |
|  | Yes | 5.4(0.8-20.8; n=33) | 13.6(7.4-23.3; n=83) | 22.5(16.0-30.6; n=137) | 21.3(14.8-29.6; n=130) | 34.5(28.2-41.4; n=210) |
| 1. No response =<5% 2. Including hospital treatment/psychiatric/mental health services and GP | | | | | | |
