# Supplementary material for: Exploring the Views of Young People, Including Those With a History of Self-Harm, on the Use of Their Routinely Generated Data for Mental Health Research: Web-Based Cross-Sectional Survey Study
Source: JMIR Ment Health. 2025 Mar 12;12:e60649. doi: 10.2196/60649 (PMC11947630; doi:10.2196/60649)
Supplement: Multimedia Appendix 6 [file mental_v12i1e60649_app6.docx]

Supplementary Table 3b Distribution of answers to the question ‘When thinking about sharing mental health data, to what extent do you disagree or agree with the following statements?’ from the SH group stratified by having contact to health services following self-harm %(95% CI;n=)^a^

|  | Contact to health services^b^ | Extremely likely | Somewhat likely | Neither likely nor unlikely | Somewhat unlikely | Extremely unlikely |
| --- | --- | --- | --- | --- | --- | --- |
| People should have the right to opt out of mental health data sharing | No | 77.4(73.8-80.6; n=605) | 14.2(8.5-22.4; n=111) | 2.7(0.0-23.1; n=21) | 0.5(2.1-60.8; n=4) | 0.1(10.7-94.6; n=1) |
|  | Yes | 77.3(73.2-81.0; n=471) | 14.9(8.6-24.3; n=91) | 2.3(0.1-29.8; n=14) | 0.3(4.7-80.4; n=2) | 0.2(10.7-94.6; n=1) |
| Mental health data should be used to understand more about mental illnesses | No | 66.6(62.4-70.6; n=521) | 24.8(19.0-31.6; n=194) | 2.8(0.0-22.5; n=22) | 0.8(1.2-49.1; n=6) | 0.6(1.6-54.3; n=5) |
|  | Yes | 71.3(66.7-75.4; n=434) | 20.9(14.4-29.2; n=127) | 2.3(0.1-29.8; n=14) | 0.8(1.5-54.5; n=5) | 0.3(4.7-80.4; n=2) |
| It is important that mental health data is held by an organisation I trust | No | 66.5(62.2-70.5; n=520) | 23.0(17.2-30.0; n=180) | 4.7(0.7-18.6; n=37) | 0.5(2.1-60.8; n=4) | 0.0(0.0-0.0; n=0) |
|  | Yes | 67.3(62.5-71.8; n=410) | 21.3(14.8-29.6; n=130) | 5.4(0.8-20.8; n=33) | 0.5(2.9-69.3; n=3) | 0.5(2.9-69.3; n=3) |
| People should be asked for consent every time a researcher wants to use their data in a new project | No | 56.9(52.2-61.5; n=445) | 24.3(18.5-31.1; n=190) | 10.2(4.9-19.5; n=80) | 3.3(0.1-20.9; n=26) | 0.5(2.1-60.8; n=4) |
|  | Yes | 53.9(48.3-59.3; n=328) | 26.1(19.6-33.8; n=159) | 8.4(2.8-20.4; n=51) | 5.6(0.9-20.7; n=34) | 1.0(1.1-49.3; n=6) |
| It should be impossible for mental health data to be linked back to the person who provided it | No | 38.6(33.1-44.4; n=302) | 29.8(24.1-36.2; n=233) | 14.7(9.0-22.8; n=115) | 9.8(4.5-19.3; n=77) | 2.4(0.0-24.4; n=19) |
|  | Yes | 37.8(31.5-44.4; n=230) | 28.4(22.0-35.8; n=173) | 15.9(9.6-25.1; n=97) | 10.7(4.7-21.4; n=65) | 2.5(0.0-28.7; n=15) |
| Researchers studying mental health should have advisors with personal experiences of mental health conditions | No | 29.9(24.2-36.3; n=234) | 39.3(33.8-45.0; n=307) | 19.9(14.2-27.3; n=156) | 4.7(0.7-18.6; n=37) | 0.8(1.2-49.1; n=6) |
|  | Yes | 37.3(31.0-44.0; n=227) | 31.5(25.1-38.7; n=192) | 19.7(13.2-28.2; n=120) | 6.1(1.2-20.4; n=37) | 0.7(2.1-61.0; n=4) |
| I would be less likely to access NHS mental health services if I knew my data might be shared with researchers | No | 12.1(6.6-20.8; n=95) | 22.5(16.7-29.5; n=176) | 28.4(22.7-34.9; n=222) | 20.7(14.9-27.9; n=162) | 10.7(5.3-19.9; n=84) |
|  | Yes | 10.8(4.9-21.5; n=66) | 20.0(13.5-28.4; n=122) | 24.1(17.6-32.0; n=147) | 24.0(17.5-31.9; n=146) | 15.9(9.6-25.1; n=97) |
| 1. No response =<5% 2. Including hospital treatment/psychiatric/mental health services and GP | | | | | | |
