# Supplementary material for: Exploring the Views of Young People, Including Those With a History of Self-Harm, on the Use of Their Routinely Generated Data for Mental Health Research: Web-Based Cross-Sectional Survey Study
Source: JMIR Ment Health. 2025 Mar 12;12:e60649. doi: 10.2196/60649 (PMC11947630; doi:10.2196/60649)
Supplement: Multimedia Appendix 7 [file mental_v12i1e60649_app7.docx]

Supplementary Table 4a Distribution of answers to the question ‘How would the following measures change the likelihood that you would be willing to share your mental health data for research purposes’ stratified by SH^a^ and NoSH^b^ groups %(95% CI;n).

|  |  | Extremely likely | Somewhat likely | Neither likely nor unlikely | Somewhat unlikely | Extremely unlikely |
| --- | --- | --- | --- | --- | --- | --- |
| My name would be removed from all data used for research purposes | SH | 42.6(38.7-46.6; n=617) | 33.1(28.9-37.5; n=479) | 9.9(5.8-16.3; n=144) | 4.0(0.8-13.7; n=58) | 5.6(2.0-13.7; n=81) |
|  | NoSH | 37.5(29.0-46.9; n=119) | 37.9(29.3-47.2; n=120) | 12.6(4.8-27.7; n=40) | 3.8(0.0-34.9; n=12) | 4.4(0.0-32.5; n=14) |
|  | ALL | 41.7(38.1-45.4; n=736) | 33.9(30.2-37.9; n=599) | 10.4(6.6-16.0; n=184) | 4.0(1.0-12.4; n=70) | 5.4(2.0-12.6; n=95) |
| I would be asked for permission every time someone wanted to look at my data | SH | 29.1(24.9-33.8; n=422) | 41.1(37.1-45.2; n=595) | 12.8(8.5-18.7; n=185) | 8.6(4.6-15.4; n=125) | 5.0(1.5-13.6; n=72) |
|  | NoSH | 23.3(14.6-34.9; n=74) | 42.0(33.6-50.8; n=133) | 17.0(8.6-30.2; n=54) | 9.1(2.1-27.0; n=29) | 6.0(0.4-29.0; n=19) |
|  | ALL | 28.1(24.2-32.3; n=496) | 41.2(37.7-44.9; n=728) | 13.5(9.6-18.7; n=239) | 8.7(5.0-14.6; n=154) | 5.2(1.8-12.5; n=91) |
| People using my data would have to do a special training course before they could access it | SH | 28.0(23.7-32.7; n=405) | 38.7(34.6-42.9; n=560) | 14.8(10.5-20.5; n=215) | 6.5(2.7-14.0; n=94) | 6.5(2.7-14.0; n=94) |
|  | NoSH | 22.1(13.4-33.9; n=70) | 40.4(31.9-49.4; n=128) | 18.9(10.4-31.5; n=60) | 8.5(1.7-27.1; n=27) | 5.7(0.3-29.5; n=18) |
|  | ALL | 26.9(23.0-31.2; n=475) | 39.0(35.3-42.7; n=688) | 15.6(11.6-20.5; n=275) | 6.9(3.3-13.3; n=121) | 6.3(2.8-13.0; n=112) |
| My data would be part of a huge database containing data from many hundreds of other people | SH | 25.8(21.5-30.6; n=374) | 38.5(34.4-42.7; n=557) | 17.3(13.0-22.7; n=251) | 7.8(3.8-14.8; n=113) | 6.4(2.6-14.0; n=93) |
|  | NoSH | 23.0(14.3-34.6; n=73) | 40.1(31.6-49.2; n=127) | 18.6(10.1-31.3; n=59) | 9.5(2.4-27.0; n=30) | 6.0(0.4-29.0; n=19) |
|  | ALL | 25.3(21.4-29.7; n=447) | 38.8(35.1-42.5; n=684) | 17.6(13.6-22.4; n=310) | 8.1(4.4-14.2; n=143) | 6.3(2.8-13.0; n=112) |
| I would not be able to withdraw my data in the future | SH | 8.2(4.2-15.1; n=119) | 7.5(3.5-14.6; n=108) | 13.5(9.2-19.3; n=195) | 22.9(18.6-27.9; n=332) | 42.4(38.5-46.4; n=614) |
|  | NoSH | 6.6(0.7-28.3; n=21) | 8.8(1.9-27.1; n=28) | 17.4(8.9-30.4; n=55) | 21.8(13.1-33.6; n=69) | 41.0(32.6-50.0; n=130) |
|  | ALL | 7.9(4.2-14.0; n=140) | 7.7(4.0-13.9; n=136) | 14.2(10.2-19.3; n=250) | 22.7(18.8-27.2; n=401) | 42.2(38.6-45.8; n=744) |
| My data might be matched with other information about me, like records from my school | SH | 7.7(3.7-14.7; n=111) | 13.2(8.9-19.0; n=191) | 16.9(12.6-22.3; n=245) | 24.1(19.8-29.0; n=349) | 33.3(29.1-37.7; n=482) |
|  | NoSH | 6.9(0.8-28.0; n=22) | 13.2(5.3-28.0; n=42) | 16.4(8.0-29.8; n=52) | 21.5(12.8-33.4; n=68) | 38.2(29.6-47.5; n=121) |
|  | ALL | 7.5(3.9-13.8; n=133) | 13.2(9.3-18.4; n=233) | 16.8(12.9-21.7; n=297) | 23.6(19.7-28.1; n=417) | 34.2(30.4-38.1; n=603) |
| I would have no control over what my data was used for in the future | SH | 7.2(3.3-14.4; n=104) | 8.0(4.0-14.9; n=116) | 11.6(7.4-17.7; n=168) | 22.9(18.6-27.9; n=332) | 45.7(41.9-49.6; n=662) |
|  | NoSH | 6.9(0.8-28.0; n=22) | 11.0(3.5-27.2; n=35) | 9.8(2.6-27.0; n=31) | 24.3(15.5-35.6; n=77) | 44.2(35.9-52.8; n=140) |
|  | ALL | 7.1(3.5-13.5; n=126) | 8.6(4.8-14.5; n=151) | 11.3(7.4-16.7; n=199) | 23.2(19.2-27.6; n=409) | 45.4(42.0-49.0; n=802) |
| 1. Self-harm group 2. No self-harm group 3. No response <=5% | | | | | | |
