# Supplementary material for: Exploring the Views of Young People, Including Those With a History of Self-Harm, on the Use of Their Routinely Generated Data for Mental Health Research: Web-Based Cross-Sectional Survey Study
Source: JMIR Ment Health. 2025 Mar 12;12:e60649. doi: 10.2196/60649 (PMC11947630; doi:10.2196/60649)
Supplement: Multimedia Appendix 9 [file mental_v12i1e60649_app9.docx]

Supplementary Table 5a Distribution of answers to the question ‘In your opinion, how trustworthy are the following organisations when it comes to storing and using mental health data for research?’ stratified by SH^a^ and NoSH^b^ groups %(95% CI;n)^c^.

| Organisation | | Very Trustworthy | Somewhat trustworthy | Neither | Somewhat untrustworthy | Not at all trustworthy |
| --- | --- | --- | --- | --- | --- | --- |
| The NHS | SH | 34.1(30.0-38.5; n=494) | 43.9(40.0-47.8; n=635) | 11.7(7.5-17.8; n=170) | 6.5(2.7-14.0; n=94) | 2.1(0.0-17.3; n=30) |
|  | NoSH | 48.9(40.8-57.0; n=155) | 37.5(29.0-46.9; n=119) | 11.0(3.5-27.2; n=35) | 0.9(2.7-69.6; n=3) | 0.6(4.6-80.5; n=2) |
|  | All | 36.8(33.1-40.6; n=649) | 42.7(39.2-46.3; n=754) | 11.6(7.7-17.0; n=205) | 5.5(2.1-12.6; n=97) | 1.8(0.0-16.1; n=32) |
| Mental health charities | SH | 36.0(32.0-40.4; n=522) | 43.4(39.5-47.4; n=629) | 12.9(8.6-18.8; n=187) | 3.6(0.6-14.0; n=52) | 1.7(0.0-19.6; n=24) |
|  | NoSH | 40.7(32.2-49.7; n=129) | 43.5(35.2-52.2; n=138) | 11.0(3.5-27.2; n=35) | 1.6(1.2-55.2; n=5) | 1.3(1.8-61.5; n=4) |
|  | All | 36.9(33.2-40.7; n=651) | 43.5(39.9-47.1; n=767) | 12.6(8.7-17.8; n=222) | 3.2(0.5-12.8; n=57) | 1.6(0.0-17.4; n=28) |
| Universities | SH | 20.0(15.6-25.1; n=289) | 51.0(47.4-54.7; n=739) | 19.3(15.0-24.6; n=280) | 4.5(1.1-13.6; n=65) | 1.3(0.1-22.8; n=19) |
|  | NoSH | 29.7(20.9-40.1; n=94) | 50.8(42.8-58.7; n=161) | 13.2(5.3-28.0; n=42) | 3.8(0.0-34.9; n=12) | 0.6(4.6-80.5; n=2) |
|  | All | 21.7(17.7-26.2; n=383) | 51.0(47.7-54.3; n=900) | 18.2(14.3-23.0; n=322) | 4.4(1.2-12.4; n=77) | 1.2(0.1-21.0; n=21) |
| The UK government | SH | 6.8(3.0-14.2; n=99) | 24.1(19.8-29.0; n=349) | 24.7(20.3-29.5; n=357) | 24.7(20.4-29.6; n=358) | 17.7(13.3-23.0; n=256) |
|  | NoSH | 9.5(2.4-27.0; n=30) | 33.8(25.1-43.6; n=107) | 22.1(13.4-33.9; n=70) | 21.8(13.1-33.6; n=69) | 11.4(3.8-27.3; n=36) |
|  | All | 7.3(3.7-13.6; n=129) | 25.8(21.9-30.2; n=456) | 24.2(20.3-28.6; n=427) | 24.2(20.3-28.6; n=427) | 16.5(12.6-21.4; n=292) |
| Devolved governments (e.g. Scottish, Welsh or Northern Irish governments) | SH | 5.1(1.6-13.6; n=74) | 21.2(16.9-26.3; n=307) | 46.7(42.9-50.5; n=676) | 14.3(10.0-20.0; n=207) | 8.9(4.8-15.5; n=129) |
|  | NoSH | 8.2(1.5-27.2; n=26) | 24.0(15.2-35.4; n=76) | 46.7(38.5-55.0; n=148) | 12.9(5.0-27.9; n=41) | 5.7(0.3-29.5; n=18) |
|  | All | 5.7(2.3-12.7; n=100) | 21.7(17.7-26.2; n=383) | 46.7(43.2-50.2; n=824) | 14.1(10.1-19.1; n=248) | 8.3(4.6-14.3; n=147) |
| Your local authority/council | SH | 5.0(1.5-13.6; n=73) | 27.1(22.8-31.8; n=392) | 32.3(28.1-36.7; n=467) | 21.3(17.0-26.4; n=309) | 10.4(6.2-16.7; n=151) |
|  | NoSH | 6.6(0.7-28.3; n=21) | 37.9(29.3-47.2; n=120) | 34.4(25.7-44.2; n=109) | 12.6(4.8-27.7; n=40) | 6.0(0.4-29.0; n=19) |
|  | All | 5.3(2.0-12.6; n=94) | 29.0(25.2-33.2; n=512) | 32.6(28.8-36.7; n=576) | 19.8(15.8-24.4; n=349) | 9.6(5.8-15.3; n=170) |
| Private companies | SH | 3.4(0.5-14.2; n=49) | 18.2(13.9-23.5; n=264) | 32.1(27.9-36.6; n=465) | 28.5(24.2-33.1; n=412) | 15.4(11.1-21.0; n=223) |
|  | NoSH | 2.8(0.2-40.5; n=9) | 21.1(12.5-33.2; n=67) | 28.4(19.6-39.0; n=90) | 29.7(20.9-40.1; n=94) | 16.4(8.0-29.8; n=52) |
|  | All | 3.3(0.5-12.7; n=58) | 18.8(14.8-23.5; n=331) | 31.4(27.6-35.5; n=555) | 28.7(24.8-32.9; n=506) | 15.6(11.6-20.5; n=275) |
| 1. Self-harm group 2. No self-harm group 3. No response <=5% | | | | | | |
