# Supplementary material for: Exploring the Views of Young People, Including Those With a History of Self-Harm, on the Use of Their Routinely Generated Data for Mental Health Research: Web-Based Cross-Sectional Survey Study
Source: JMIR Ment Health. 2025 Mar 12;12:e60649. doi: 10.2196/60649 (PMC11947630; doi:10.2196/60649)
Supplement: Multimedia Appendix 10 [file mental_v12i1e60649_app10.docx]

Supplementary Table 5b Distribution of answers to the question ‘‘In your opinion, how trustworthy are the following organisations when it comes to storing and using mental health data for research?’ from the SH group stratified by having contact to health services following self-harm %(95% CI;n=)^a^

| Organisation | Contact to health services^b^ | Very Trustworthy | Somewhat trustworthy | Neither | Somewhat untrustworthy | Not at all trustworthy |
| --- | --- | --- | --- | --- | --- | --- |
| The NHS | No | 35.3(29.7-41.3; n=276) | 42.3(37.0-47.9; n=331) | 12.7(7.1-21.2; n=99) | 6.6(1.9-18.1; n=52) | 1.4(0.3-33.9; n=11) |
|  | Yes | 32.5(26.1-39.6; n=198) | 46.6(40.7-52.6; n=284) | 10.2(4.3-21.2; n=62) | 6.4(1.4-20.3; n=39) | 2.6(0.0-27.7; n=16) |
| Mental health charities | No | 36.1(30.5-42.0; n=282) | 45.1(39.9-50.5; n=353) | 12.5(7.0-21.1; n=98) | 3.2(0.1-21.2; n=25) | 1.2(0.6-38.5; n=9) |
|  | Yes | 36.1(29.9-42.9; n=220) | 41.4(35.3-47.7; n=252) | 13.8(7.5-23.4; n=84) | 4.3(0.3-22.2; n=26) | 2.0(0.2-32.7; n=12) |
| Universities | No | 18.8(13.0-26.3; n=147) | 55.2(50.4-60.0; n=432) | 18.2(12.4-25.7; n=142) | 4.3(0.5-19.0; n=34) | 1.0(0.7-41.4; n=8) |
|  | Yes | 21.7(15.2-29.9; n=132) | 46.1(40.2-52.2; n=281) | 21.0(14.5-29.3; n=128) | 4.6(0.4-21.6; n=28) | 1.3(0.7-41.7; n=8) |
| The UK government | No | 5.5(1.1-18.2; n=43) | 24.8(19.0-31.6; n=194) | 24.0(18.3-30.9; n=188) | 26.2(20.4-32.9; n=205) | 17.4(11.6-25.0; n=136) |
|  | Yes | 8.7(3.1-20.5; n=53) | 23.0(16.5-31.0; n=140) | 25.9(19.5-33.6; n=158) | 22.5(16.0-30.6; n=137) | 18.2(11.8-26.9; n=111) |
| Devolved governments (e.g. Scottish, Welsh or Northern Irish governments) | No | 5.0(0.8-18.4; n=39) | 20.7(14.9-27.9; n=162) | 47.8(42.7-53.0; n=374) | 14.8(9.2-22.9; n=116) | 8.6(3.5-18.6; n=67) |
|  | Yes | 5.6(0.9-20.7; n=34) | 21.7(15.2-29.9; n=132) | 45.6(39.7-51.7; n=278) | 13.8(7.5-23.4; n=84) | 9.2(3.5-20.7; n=56) |
| Your local authority/council | No | 4.2(0.4-19.2; n=33) | 28.1(22.4-34.6; n=220) | 32.7(27.1-38.9; n=256) | 22.0(16.2-29.1; n=172) | 10.2(4.9-19.5; n=80) |
|  | Yes | 6.2(1.3-20.4; n=38) | 25.5(19.0-33.2; n=155) | 32.2(25.8-39.3; n=196) | 20.7(14.2-29.0; n=126) | 10.2(4.3-21.2; n=62) |
| Private companies | No | 3.2(0.1-21.2; n=25) | 17.9(12.1-25.5; n=140) | 32.6(27.0-38.8; n=255) | 29.5(23.8-35.9; n=231) | 15.0(9.3-23.0; n=117) |
|  | Yes | 3.9(0.2-22.8; n=24) | 19.2(12.7-27.8; n=117) | 31.9(25.5-39.0; n=194) | 27.6(21.1-35.1; n=168) | 14.9(8.6-24.3; n=91) |
| 1. No response =<5% 2. Including hospital treatment/psychiatric/mental health services and GP | | | | | | |
